# The prevalence of burnout syndrome among resident physicians in Syria

**DOI:** 10.1186/s12995-019-0250-0

**Published:** 2019-12-10

**Authors:** Bahaa Aldin Alhaffar, Ghadir Abbas, Alaa Aldin Alhaffar

**Affiliations:** 1 Department of Periodontology, Faculty of Dental Medicine, Damascus University, Damascus, Syria; 2 Department of Psychiatry, Faculty of Medicine, Damascus University, Damascus, Syria; 3 Department of Cardiology, Yousef al Aazmah Hospital, Damascus, Syria; 4 Al-Mowasat University Hospital, Omar Bin Abdulaziz, Damascus, Syria

**Keywords:** Burnout, Residents, Physicians, Syria, War

## Abstract

**Introduction:**

Burnout syndrome is a work-related chronic stress that is described as emotional exhaustion, a decreased sense of personal accomplishment, and depersonalization. it has been considered an important problem especially among workers in the health sector.

**Objectives:**

The aim of this research was to study the prevalence of burnout among Syrian residents during the Syrian crisis, which started 9 years ago, and to assess the factors related to burnout syndrome.

**Methods:**

A cross-sectional study was conducted to assess the levels of burnout syndrome; data were collected from residents doing their residency in 12 different hospitals spread over 8 governorates in Syria. A web-based Arabic version of Maslach Burnout Inventory questionnaire was used. The final sample size was 3350 residents from different specialties. SPSS V.22 was used to analyses the data using descriptive and inferential statistics.

**Results:**

(93.75%) had a high level in at least one of the three domains of the burnout index, and (19.3%) of the residents had a high level of burnout in all three domains. Significant relation was found between gender, age group and affiliated authority variables and the levels of burnout. However no significant relation was found between burnout and the specialties or geographic variables. Males, residents in Ministry of Defense, and emergency medicine residents had the highest levels of burnout.

**Conclusion:**

High levels of burnout was found among residents during the Syrian crisis in comparison with other studies, which highlights the role of the current situation in raising workload on the Syrian residents.

## Introduction

Burnout syndrome was first described in 1974 among healthcare volunteers [1]. It is defined as the response to work-related processes of chronic stress. It has been considered as an important health problem in the past decade. According to Maslach, Burnout syndrome consists of three components: emotional exhaustion (emotional overextension and exhaustion), depersonalization (negative, callous, and detached responses to others), and reduced personal accomplishment (feelings of competence and achievement in one’s work) [2, 3]. Quantitative job demands like experienced workload and time pressure are strongly and consistently related to burnout, particularly the exhaustion dimension, qualitative job demands like role conflict and role ambiguity are also significantly correlated with burnout [4].

Work-related stress among healthcare professionals has become a serious health problem.

For workers and the world economy [5], and burnout among training and practicing physicians have reached epidemic levels with a prevalence that approximates 50% [6, 7]. There are four main factors associated with primary health care workers burnout: 1) a lack of control over work conditions and decision making; 2) time pressure such that there was a perception by physicians that they were only valued for their productivity; 3) a chaotic and inefficient work environment that inappropriately used physicians to do clerical and other mundane tasks; and 4) a lack of alignment among physicians and executives regarding values, mission, purpose, and compensation [5]. Specific stresses that may predispose residents to burnout: excessive demands that reduce the quality of care, long working hours, numerous work shifts and sleep deprivation, the need to deal with suffering and death and the fear of making mistakes, and more [8, 9]. Lack of autonomy, competitiveness, new expectations, inadequate support from supervisors and irregular work schedules are other problems of residency that correlate with burnout [10].

Burnout was found to be a significant predictor of the following physical conditions: hypercholesterolemia, type 2 diabetes, coronary heart disease, hospitalization due to cardiovascular disorder, musculoskeletal pain, changes in pain experiences, prolonged fatigue, headaches, gastrointestinal issues, respiratory problems, severe injuries and early mortality age, while psychological effects include: insomnia, depressive symptoms, use of psychotropic and antidepressant medications, hospitalization for mental disorders and psychological ill-health symptoms, in addition to a correlation between burnout and suicidal thinking [11]. Therefore, this is a public health problem with disastrous consequences that must be prevented in the workplace.

On the other hand, The Syrian crisis began in 2011 and has had serious consequences on all of the Syrians. There are roughly 13 million people currently in need of medical services, and the requirements necessary to help these people have exceeded the available resources. WHO -World Health Organization- has only received 1/3 of the funding needed to implement the Humanitarian Activities Plan for 2016 [12]. The negative side effects of wars and conflicts on mental health are well documented [13]. The Syrian long-lasting crisis and the presence of conflict took a wide range of influence and affected every aspect of life, such as health care and education which has reflected on every person and caused a deterioration of living conditions [14–16]. This too adds to the stresses affecting medical residents and doctors who aside from suffering because of the war, had to carry the burdens of healthcare deterioration by making extra efforts to fulfill their roles as care providers.

The purposes of our study were to investigate the prevalence of burnout syndrome among resident physicians of all specialties in Syria, and to identify the main demographic factors associated with Burnout Syndrome.

## Material and methods

A cross-sectional study was conducted on a large sample of resident physicians training in Syrian hospitals affiliated to 3 residency programs in Syria: Ministry of Higher Education (MHE), Ministry of Health (MH), and Ministry of Defense (MD). We had also collected data from residents working in private hospitals in Syria. Data were collected from 12 hospitals located in 8 governorates (Damascus, Aleppo, Lattakia, Tartous, Daraa, Rif Dimashq, Hamah, Homs), other governorates were not reachable during the time of data collection. All of the included doctors were current medical residents who spent at least one year in a residency program approved by the Syrian Commission of the Medical Specialties. We excluded residents who did not complete the research questionnaire, or had spent less than one complete year in residency, doctors who had finished their residency were also excluded.

Residents were contacted by Email, the purpose of the study was thoroughly explained and participation was voluntary, the data was collected during the month of July 2018.

### Material

We designed a special survey to collect the data, all residents of all specialties and hospitals were invited to participate. The survey consisted two parts: the first part was demographic data sheet that was designed especially for the purpose of this study, containing questions about age, gender, governorate, hospital of residency, residency program, the resident’s specialty, and the residency year. The second part was the Maslach Burnout Inventory - Human Services Survey (MBI-HSS), we used an Arabic validated version [17], MBI-HSS is structured with 22 questions subdivided into the areas of emotional exhaustion, depersonalization and professional achievement. These three domains were evaluated as the following: for EE (low: < 13, moderate: 14–26, high > 27), for DP (low: < 5, moderate 6–9, and high > 9), for PA (low > 40, moderate 34–39, high: < 33). Also, further dichotomization was used for the three burnout scores (high burnout versus no high burnout).

### Statistical analysis

We contacted (3837) resident physicians across Syria, (3350) residents completed the questionnaire and therefore, the response rate was (87.3%).

Data were analyzed using SPSS v.22 for descriptive data (percent, mean, standard deviation), and inferential statistics. The following tests were used to compare proportions of high levels of burnout: T-test, ANOVA test, Pearson’s correlations, and the significance level was defined as *p* < 0.05.

## Results

The final sample size was (3350), Table 1 represents the distribution of residents according to demographic and work-related variables. (44.1%) of the residents were females and (55.9%) were males, age ranged between 21 and 35 years, age was categorized into three groups 21–25 (34%), 26–30 (59.4%) and 31–35 (6.5%). Participants were doing their residency in 8 different governorates in Syria (out of 14), and almost half of them (51.3%) were located in Damascus city which is the capital and has the main functional hospitals in Syria. Residents were distributed among 4 authorities and each one has a residency program as the following: (MHE) ministry of higher education (39.1%), (MH) ministry of health (47.1%), (MD) ministry of defense (11.3%), and (2.5%) were working in private hospitals (PH). Participants were training in 12 different specialties with general surgery as the most represented specialty (32.6%).

**Table 1:** Distribution of the sample according to the demographic and work-related variables

Variables		Percentage	Frequency
Gender	Females	44.10%	1477
	Males	55.90%	1873
Age group	21–25	34%	1139
	26–30	59.50%	1994
	31–35	6.50%	217
Governate	Damascus	51.30%	1718
	Aleppo	13.40%	449
	Latakia	11.90%	398
	Tartous	6.20%	207
	Daraa’	5.70%	190
	Rif Dimashq	2.30%	78
	Hamah	6%	202
	Homes	3.20%	108
Related authority	MHE	39.1%	1311
	MH	47.1%	1577
	MD	11.3%	378
	PH	2.5%	84
Academic year	First	39.10%	1311
	Second	26.50%	887
	Third	15.20%	510
	Fourth	10.70%	358
	Fifth	8.50%	284
Specialty	General Surgery	32.6%	1092
	Internal Medicine	24.2%	811
	Pediatrics	9.2%	309
	ENT	2.4%	81
	Ophthalmic	5.9%	197
	Obstetrics	5.7%	190
	Dermatology	2.4%	81
	Radiology	5.7%	190
	Oncology	2.7%	90
	Psychiatry	2.4%	81
	Emergency Medicine	4.4%	147
	Neurology	2.4%	81

*MHE* Ministry of Higher Education, *MH* Ministry of Health, *MD* Ministry of Defense, *PH* Private Hospitals, *ENT* Ear, nose and throat

Levels of emotional exhaustion, depersonalization, and personal accomplishment are displayed in Table 2, (77.9%) of the sample had a high level of (EE), (54.6%) had a high level of (DP), and (13.7%) had a low level of (PA). (19.3%) of the residents included in this study had a high level of burnout in all three domains of the index, and (93.75%) had a high level in at least one of the three.

**Table 2:** Levels of burnout for the three dimensions of the index

	EE		DP		PA	
	Percent	Frequency	Percent	Frequency	Percent	Frequency
Low level	6%	201	16.1%	539	64.5%	2160
Moderate	16.1%	539	29.3%	981	21.8%	730
High level	77.9%	2609	54.6%	1829	13.7%	458
					Percent	Frequency
High level of burnout in all domains of the index					19.3%	646
High level of burnout in at least one domain of the index					93.75%	3140

*EE* Emotional Exhaustion, *DP* Depersonalization, *PA* Personal Accomplishment

**Fig. 1:**
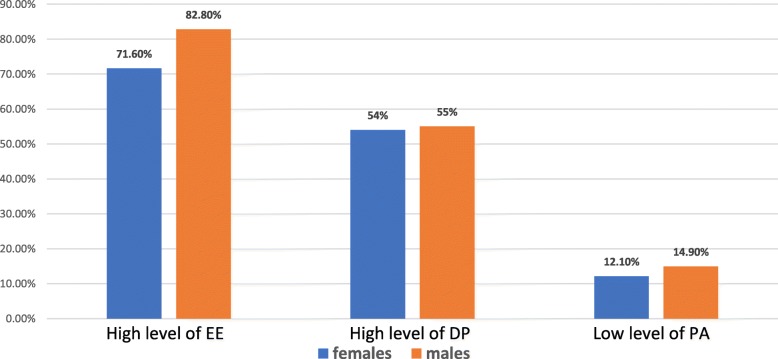
Gender and Levels of Burnout; Males had higher levels of EE, and DP, and lower levels of PA than females

Table 3 represents the percentage of the high level of each burnout components distributed according to the research different demographic variables and the results of the inferential statistical tests. Males had higher levels of EE (82.8%), and DP (55%), and lower levels of PA (14.9%) than females (71.6%), (54%), (12.1%) respectively. These results are also presented in Fig. 1. Significant relation was only found between gender and level of EE using T-test (*p* = 0.004).

**Table 3:** Research variables and statistical test

Research variables			Burnout variables			Statistical tests	
Variable		Percent	High level of EE	High level of DP	High level of PA	Statistical test	*P* value
Gender	Females	44.10%	71.6%	54%	12.1%	T-test	0.004 for EE
	Males	55.90%	82.8%	55%	14.9%		
Age group	21–25	34%	73.6%	56.1%	12.2%	ANOVA	0.037 for EE
	26–30	59.50%	79.3%	53.2%	13%		
	31–35	6.50%	86.3%	59%	27.2%		
Governate	Damascus	51.30%	77.6%	53.6%	14%	ANOVA	0.528 No significant relation
	Aleppo	13.40%	73.3%	51.1%	15.5%		
	Latakia	11.90%	72.5%	45%	17.5%		
	Tartous	6.20%	85.7%	52.3%	9.5%		
	Daraa’	5.70%	78%	56%	4.5%		
	Rif Dimashq	2.30%	80%	87.5%	12.5%		
	Hamah	6%	88.2	76.4%	11.7%		
	Homes	3.20%	72.2%	63.6%	3.8%		
Related authority	MHE	39.1%	75.5%	51.1%	16.7%	ANOVA	0.007
	MH	47.1%	77.2%	56.3%	12%		
	MD	11.3%	89.4%	63.1%	18.7%		
	PH	2.5%	65%	37.5%	20%		
Academic year	First	39.10%	77%	54.9%	14.5%	ANOVA	0.067 no significant relation
	Second	26.50%	68.6%	60.6%	8.9%		
	Third	15.20%	77.7%	37.2%	17.6%		
	Fourth	10.70%	80.8%	55.5%	8.3%		
	Fifth	8.50%	89.2%	64.2%	25%		
Specialty	General Surgery	32.6%	81.4%	58%	14.8%	ANOVA	0.066 no significant relation
	Internal Medicine	24.2%	76.2%	54.5%	14.6%		
	Pediatric	9.2%	80%	45%	15%		
	ENT	2.4%	78%	40%	12%		
	Ophthalmic	5.9%	77.4%	64%	19.3%		
	Obstetrics	5.7%	76.9%	60%	11.5%		
	Dermatology	2.4%	77%	56%	13.2%		
	Radiology	5.7%	64.6%	45%	5.8%		
	Oncology	2.7%	66%	45%	3.4%		
	Psychiatry	2.4%	66.6%	60%	3.4%		
	Emergency Medicine	4.4%	89%	34%	12.3%		
	Neurology	2.4%	85%	50%	4.9%		

*MHE* ministry of higher education, *MH* ministry of health, *MD* ministry of defense, *PH* privet hospital, *EE* Emotional Exhaustion, *DP* Depersonalization, *PA* Personal Accomplishment, *ENT* Ear, nose and throat

**Fig. 2:**
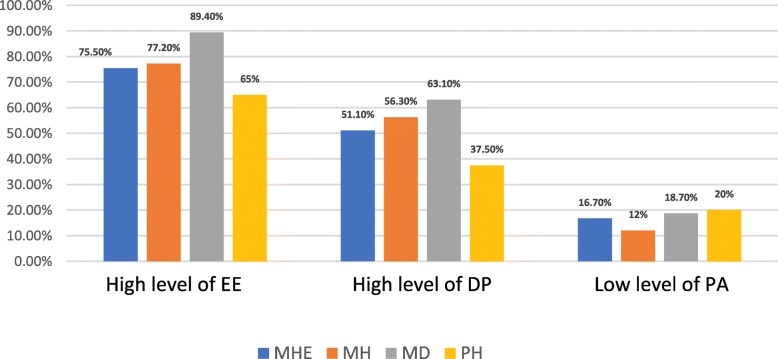
Residency Program and Levels of Burnout; Residents working in the ministry of defense’s hospitals (MD), had the highest rates of high EE and DP. while residents working in private hospitals had the highest rates of low PA

Residents working in the ministry of defense’s hospitals (MD), had the highest rates of high EE (89.4%) and DP (63.1%). while residents working in private hospitals had the highest rates of low PA (Fig. 2). ANOVA test showed a significant relation between the levels of burnout components and the residency program affiliated authority (*p* = .007).

**Fig. 3:**
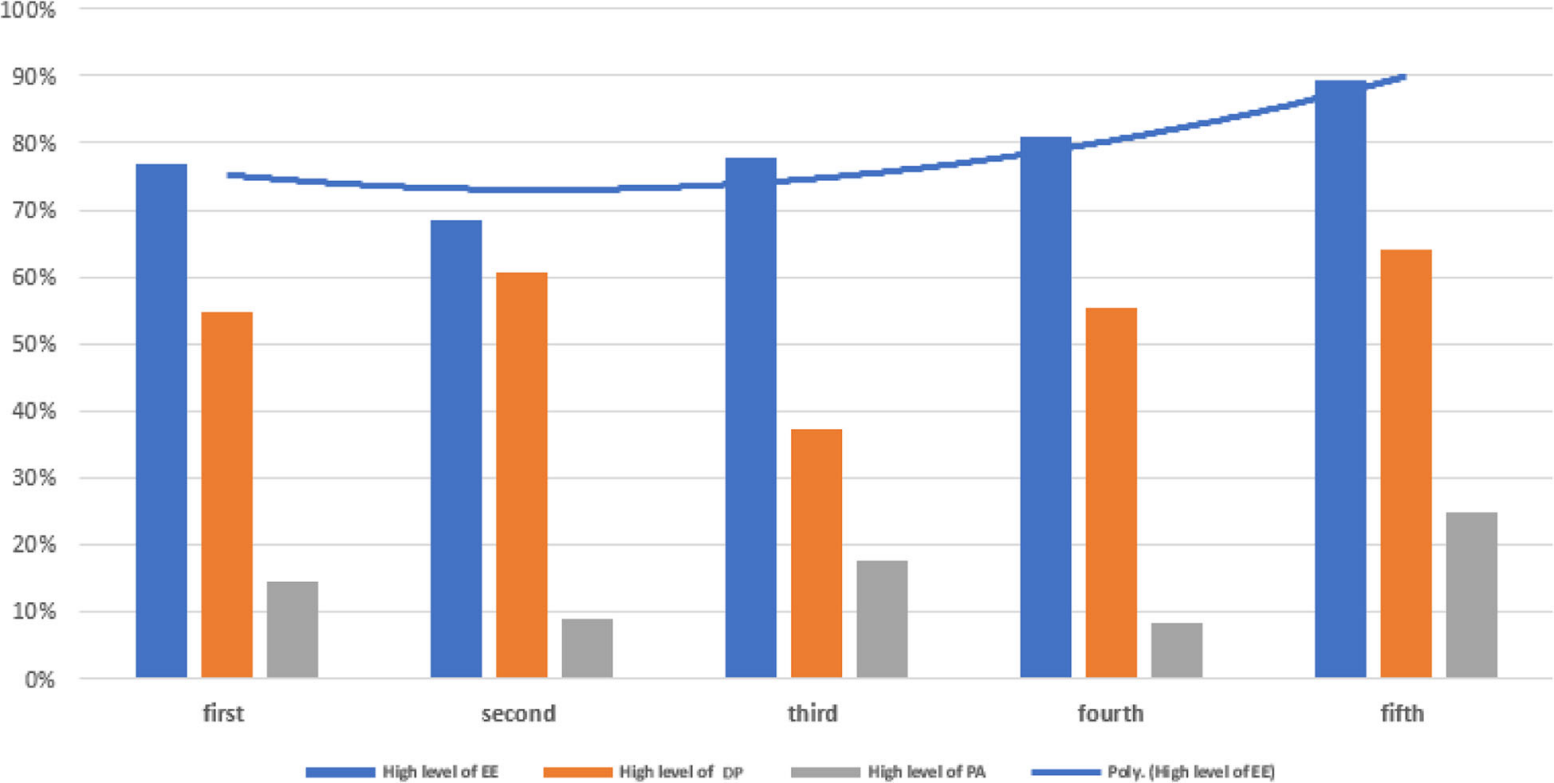
Academic Year and Levels of Burnout; Overall levels of burnout tend to be higher as residency years increase

Physicians in their fifth year of residency had the highest percentage of high EE and DP and low PA, however no significant relation was found between those two variables (*p* = .067). Moreover, Fig. 3 demonstrate that overall levels of burnout tend to be higher as residency years increase.

When comparing between specialties, no significant relation was found between that variable and burnout levels. Although, general surgery and emergency medicine residents had the highest level of EE (89%).

**Fig. 4:**
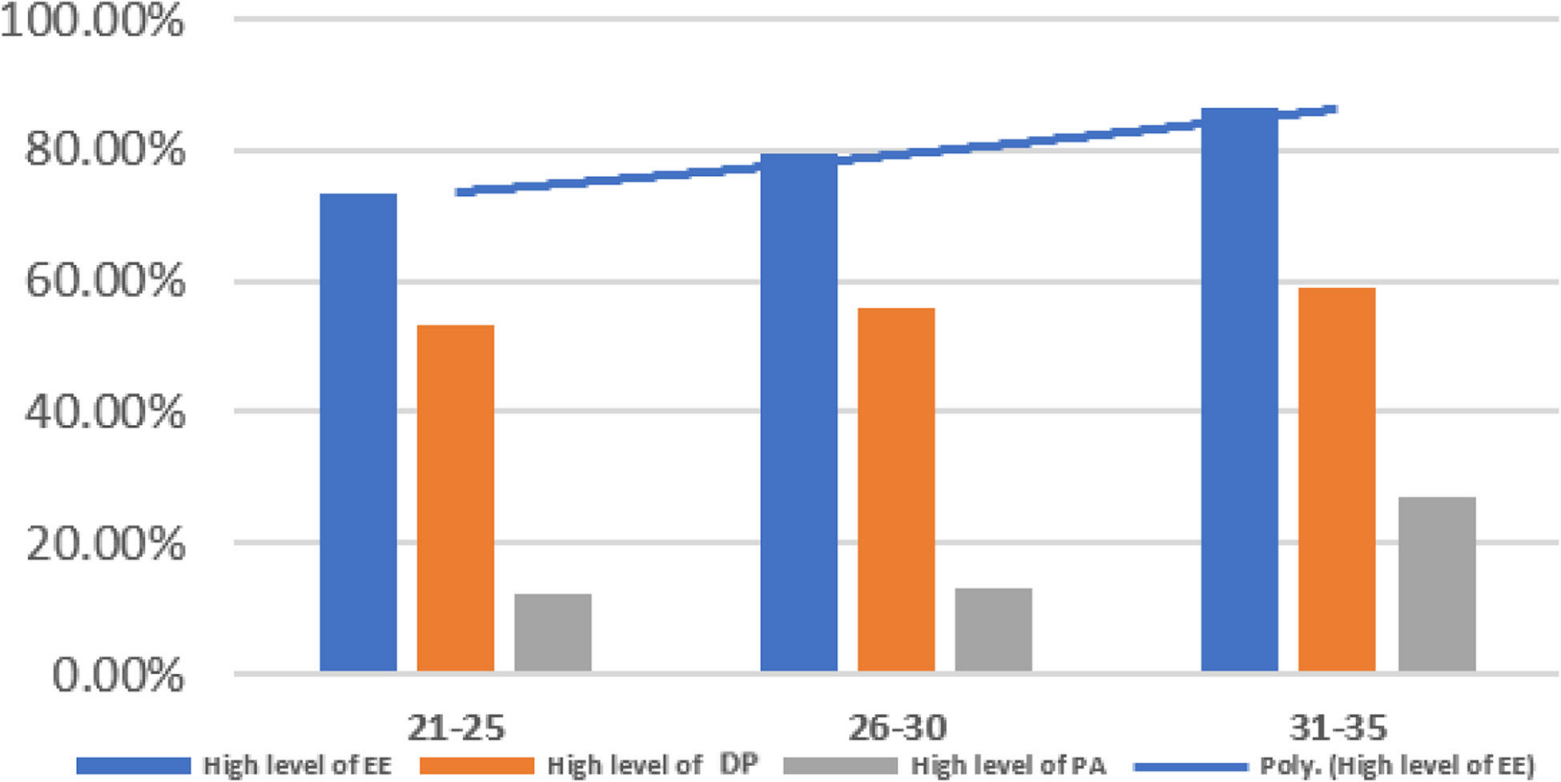
Age and Level of Burnout; higher level of burnout is found with older age group

Third age group (31–35 years) had the highest rates of burnout on all of the three domains: EE, DP and PA (86.3%), (59%) and (27.2%) respectively. ANOVA test showed significant relation between age group and level of Emotional Exhaustion (*p* = .037). Figure 4 presents that higher level of burnout can be found with older age group.

For the geographical distribution, Hamah had the highest level of EE (88.2%), Rif Dimashq had the highest level of DP (87.5%), and latakia had the highest level of PA (17.5%), No significant relation was found between the geographical governorate and the burnout variables (*p* = .528).

## Discussion

The Syrian crisis which started eight years ago, was considered the worst humanitarian crisis in the twenty-first century. it has tragically affected all the aspect of Syrian people lives, it has destroyed the economic and educational systems and had devastating impact on the health system to the point it became both very hard and dangerous for health workers and medical professionals to respond to the growing needs of the population [18, 19]. Health care providers in Syria are currently working in an environment where there is a high rate of mortality and spreading of illnesses and diseases [20]. In addition to low medical equipment and medications shortage, several health facilities were completely destroyed. Moreover, almost half of the medical doctors and professionals have escaped Syria during the years of crisis. All of this made it especially hard for the remaining doctors and health worker to respond for the growing needs of the community during the years of the war [21]. And that made this exploratory study of great importance, to document the levels and prevalence of Burnout in this specific time of crisis.

All the previous mentioned factors and some other related to the overall socio-economic status during the time of war in Syria contribute directly or indirectly in the high percentage of burnout syndrome found in this study, the prevalence of this syndrome among Syrian residents is considered one of the highest levels in the world compared to other studies conducted in different countries which used the same method of calculation. The prevalence of the two of three (EE, DP) subcategories of the burnout is higher than the EGPRN -European General Practice Research Network- report which revealed 12% for EE, 35% for DP, and 32% for PA [22], only PA in this study is lower than the level compared to EGPRN report and that may be referred to that the majority of the sample included in this present study are residents and this residency is part of their curriculum. In a similar study conducted in the American university of Beirut on medical residents, 80% of the residents had a high level of burnout in at least one of the three domains, and the highest level of burnout was found in the domain of emotional exhaustion (67.7%) and those results agree with the ours, with the exception that higher levels were presented here [23].

A recent systematic review indicated that the overall prevalence of burnout for all specialties is almost 35%. As for the burnout dimensions, the prevalence estimate of high DP, EE, and low PA for all specialties was 43.6%, 38,9%, and 34,4% respectively [24]. In United Arab Emirates 75.5% of the medical residents had moderate-to-high EE, 84% had high DP, and 74% had a low sense of PA. In aggregate, 70% of medical residents were considered to be experiencing at least one symptom of burnout based on a high EE score or a high DP score [25]. While 93% of the residents in our study had at least one positive dimension of burnout, reflecting again unusually high burnout levels among Syrian residents.

Fifth year residents had the highest rates of EE, DP, and PA. Aside from first year residents, burnout levels seemed to increase as physicians spent more years in residency, Although no statistically significant relation was found here in our study, many others stated the same notice [25, 26]. First year residents had higher levels of EE and DP than residents of the second and third years, this could be caused by the sudden transition between medical-student-life and resident-physician-life, especially when knowing that first year residents have the highest workloads and highest number of night shifts per month in the Syrian residency programs.

One of the unusual findings in our study was the relationship between gender and burnout. Male physicians registered higher levels of EE and DP and lower levels of PA than female physicians, with the knowledge that this difference was statistically significant only for emotional exhaustion dimension, although the opposite is usually reported by other studies [23, 27, 28]. Yet no conclusive data in the published literature, in fact one meta-analysis focused on the relationship between gender and burnout, results demonstrated that women were slightly more emotionally exhausted than men, while men are somewhat more depersonalized than women [29]. The disparity of our results may be justified by the fact that males are demanded by the social norms to provide the needs of their families which increased dramatically in the circumstances of the war, although this requires further investigation to establish the presence of such relationship.

In our research, we found significant relation between EE and DP and residency in military hospitals, that is because physicians working for those hospitals are subjected to military regulations that adds on to the list of stressors related to the years of residency. Not many published literatures discussed this matter, although one study reported no significant difference in high EE or low PA rates between military orthopedic residents and their civilian peers. However, military residents had a significantly lower rate of high DP than civilian residents [30]. Another research studied the prevalence of burnout syndrome among military physicians in brazil, and this research concluded that there was a considerable high level of burnout which were most noticeable at the domain of depersonalization [31]. Therefore, special programs should be developed to prevent burnout and to help residents and physicians to avoid burnout effects.

## Conclusion

High levels of burnout syndrome have been found among Syrian residents during the period of the Syrian crisis. Male physicians had higher emotional exhaustion, and residents working for Ministry of Defense had the highest rates of burnout syndrome. There was a significant relation between Burnout and gender, affiliated authority, and no other significant results between the other variables were found.

more studies are needed to understand the coping mechanisms in order to prevent and reduce the effects of chronic stress on Syrian physicians.

## Data Availability

The datasets analyzed during the current study are available from the corresponding author on reasonable request.
